# Degenerative Cervical Myelopathy and Spinal Cord Injury: Introduction to the Special Issue

**DOI:** 10.3390/jcm11154253

**Published:** 2022-07-22

**Authors:** Khadija Soufi, Aria Nouri, Allan R. Martin

**Affiliations:** 1 Department of Neurosurgery, University of California, Davis, CA 95817, USA; khsoufi@ucdavis.edu; 2 Division of Neurosurgery, Geneva University Hospitals, 1205 Geneva, Switzerland; arianouri9@gmail.com

Damage to the spinal cord (SC) can arise from either traumatic or non-traumatic spinal cord injury (SCI). Non-traumatic forms of SCI include degenerative cervical myelopathy (DCM) in which spinal degeneration secondary to age-related degeneration of the discs, ligaments, and vertebrae of the cervical spine causes cord compression, resulting in varying degrees of neurological dysfunction. On the other hand, traumatic spinal cord injury (tSCI) is principally due to immediate mechanical insult resulting in sudden onset motor, sensory and autonomic dysfunction, and secondary injury mechanisms resulting from the resulting inflammation. Both DCM and tSCI share similar pathological and molecular characteristics including neuro-inflammation, axonal degeneration, and alpha-motor neuron degeneration and result in similar patterns of anterograde and retrograde remodeling of synaptic pathways [1,2]. MRI-based imaging studies have found similarities in the degeneration of the dorsal and lateral columns and in degrees of remote SC pathology [1,2]. In addition, patients with either DCM or non-myelopathic SC compression are predisposed to tSCI from even a minor trauma, as the compressed SC is more vulnerable to dynamic forces and kinking, particularly in hyperextension injuries; this type of tSCI is commonly termed ‘central cord syndrome’ and presents with quadriparesis that affects upper extremities more than lower extremities [3]. The relationship between DCM and tSCI is still being elucidated in the literature and could offer a means to study SCI by assessing the large population of individuals with DCM that frequently have stable or slowly progressive disease.

Both traumatic and non-traumatic SCI are anatomically and physiologically complex pathologies that present with variable symptoms and severity including numbness, impaired hand dexterity, weakness, unsteady gait, and sphincter dysfunction [1,2]. Traditionally, physician administered outcome measures such as mJOA and Nurick, and patient reported NDI, have been used to classify DCM severity, while tSCI studies typically report ASIA Impairment Scale (AIS) and the ISNCSCI, which includes high reliability and objective interpretation of findings. However, the ISNCSCI is not sensitive to subtle SC dysfunction such as hand incoordination or gait imbalance, which are subjectively captured by DCM outcome measures (e.g., mJOA) [4]. Both pathologies impair patients’ mobility, strength, and coordination, significantly affecting patients’ quality of life, resulting in a significant healthcare burden as the leading cause of SC dysfunction. Over the past few years, there has been increased research on clinical course, diagnosis, treatment threshold, and patient outcomes which have guided the establishment of treatment and diagnosis guidelines. However, there remains significant knowledge gaps, and, as a consequence, practice guidelines have been formed with limited strength of evidence, indicating a continued need for further investigation.

The present Special Issue is dedicated to presenting current research topics in DCM and SCI in an attempt to bridge gaps in knowledge for both of the two main forms of SCI. The issue consists of fourteen studies, of which the majority were on DCM, the more common pathology, while three studies focused on tSCI. This issue includes two narrative reviews, three systematic reviews and nine original research papers. Areas of research covered include image studies, predictive modeling, prognostic factors, and multiple systemic or narrative reviews on various aspects of these conditions. These articles include the contributions of a diverse group of researchers with various approaches to studying SCI coming from multiple countries, including Canada, Czech Republic, Germany, Poland, Switzerland, United Kingdom, and the United States. 

The pathological impacts of DCM and tSCI are not limited to the SC; downstream and upstream neural pathways have been shown to significantly affect cortical volume with an increased connectivity within sensorimotor and pain related cortical regions which may affect patient perceived pain and symptom burden over time [5]. Oughourlian et al. [5] were one of the first to assess sex related differences in cerebral cortex changes, utilizing a vertex level linear model (*n* = 85). They found significant differences between male and female DCM patients, including significantly less grey matter volume (GMV) changes in females over a broader range of cortical areas compared to their male counterparts despite no differences between GMV volumetric differences amongst controls. These changes were also correlated with mJOA and in the future could be used to further understand role of sex-hormones and prognostic factors in pathogenesis of DCM. Wolf et al. [6] also found gender related differences in SC motion patterns amongst men with stenosis at the C5/C6 or C6/C7 levels and no relationship between cervical joint motion to severity of the stenosis indicating the need for further assessment of gender differences in pathological features of DCM. On assessment of outcome measures for DCM, Kadanka et al. [7], showed that the standardized 10 m walk/run test can assess motor and balance abnormalities in both classic DCM patients and non-myelopathic degenerative cervical cord compression (NMDCC) patients, which has a 40% prevalence in 60+ age groups in European/American subpopulation. This was the first study assessing such changes in NMDCC patients and the 10 m walk/run test closely correlated with mJOA, which could allow for early detection of DCM before permanent neurodegeneration occurs.

In terms of surgical prognostic factors, Wilson et al. [8] challenged the previously used parameter of age and found that frailty as scored by the MFI-5 has the largest effect size and is more likely to predict peri-operative adverse events including mortality, readmission or re-operation, length of hospital stay, and recovery location. This study utilized information from over 41,000 DCM patients who underwent a variety of surgical treatments with the majority (70.8%) of single or two-level pathology providing strong evidence to incorporate frailty tests such as MFI-5 in clinical practice instead of less reliable measures such as age.

Image-oriented research by Jentzsch et al. [9] assessed potential surgical prognostic factors found on MRI for prospectively collected data for 459 patients who had prior SCI and found that SC signal change is a significant predictor (109%) of adverse events including neurologic impairment and decreased ambulation initially and at follow-up one year later. These findings are in agreement with the 14 small (*n* < 100) prospective studies summarized by Jentzsch et al. in the paper which found further negative prognostic association between pre-operative SC signal change and post-operative clinical outcomes. The implications of this study are significant and highlight the need for further research on other imaging based prognostic factors through large prospective, long-term, and confounder-controlled studies.

Building on this concept, Ost et al. [10] explored the predictive modeling of MR imaging of 328 DCM patients and found that metrics such as cross-sectional area, eccentricity, and solidity were not correlated with mJOA disease severity, and with the variations appearing to be due to patient-specific parameters. This highlights the complexity of DCM and the need for further integrated approaches to modeling efforts. Imaging data is one of many core tenets to management and surgical decision making for DCM, however, assessing severity and progression continues to rely on physical and neurological measures. The authors additionally conclude that future efforts that utilize more complex models, normalize metrics per-patient, and assess healthy control variations could overcome the limitations of the current model used by Ost et al.

Beyond conservative management and close monitoring, surgical decompression is the main-stay treatment for DCM and a variety of surgical approaches and interventions have been utilized. Appropriate selection of surgical intervention is based on patient characteristics, disease pathology, and risk factors. Sommaruga et al. [11] compared the surgical outcomes including Bazaz dysphagia score, Nurick grade, and hospital stay between stand-alone zero-profile implants and more traditionally used cervical plating in anterior cervical discectomy and fusion. The study, consisting of 116 patients, found a shorter hospital course and operation time for stand-alone implants; however, neurologic and dysphagia outcomes were similar across both groups. This study adds to the growing literature on differences between various anterior surgical treatments.

On a similar note, Wincek et al. [12] studied repetitive transcranial magnetic stimulation (rTMS) and kinesiotherapy across an average of 5 months in 26 patients with incomplete SCI and found significant improvements including reduced upper extremity spasticity, motor unit recruitment and efferent neural transmission. These findings are a promising therapeutic method for enhancing outcomes in patients with incomplete SCI and addressing neurodegenerative changes in DCM. However, this area remains in its infancy.

Many patients with DCM present with uncommon symptoms and, due to the older age and complex anatomy of DCM involving both SC and brain, present with a variety of unexplained symptoms. Previous literature included cervical vertigo as a symptom which was discussed by Kadanka et al. [13] through a patient case series (*n* = 38) on vertigo in DCM patients which found alternate etiology, indicating the importance of appropriately assessing the symptoms that may occur in DCM and considering alternate diagnoses.

This Special Issue also includes three systematic reviews. The first of these, by Ghaffari-Rafi et al. [14], assessed the role and impact of obtaining an MRI in acute SCI on clinical outcomes and decision making. Of the 32 studies included, MR imaging frequently identified pathologies such as spinal cord compression, ligamentous injury, and epidural hematoma that altered the acute management of SCI, including the need for surgery, timing of surgery, and the surgical approach (anterior vs. posterior). MRI also showed good to excellent diagnostic accuracy for various types of ligamentous injury and epidural hematoma, but poor accuracy for fracture detection. This systematic review and meta-analysis strengthens the argument that obtaining MRI is important in cases of acute SCI, while highlighting knowledge gaps on cost-effectiveness and impact on outcomes.

Yang et al. [15] provided a comprehensive systematic review of posterior approaches to multi-level DCM, highlighting that the variation of study designs, outcomes, and limited direct comparison of techniques has led to lack of high-level evidence to guide surgical approach to management of DCM. Amongst the limited studies that directly compared surgical techniques, there were many contradictory findings, emphasizing the need for future RCT or prospective multi-center studies, which are currently underway in the UK with POLYFIX-DCM trial (Posterior LaminectomY and FIXation for DCM).

Lannon et al. [16] summarized the clinical presentation, treatment, and natural history of DCM in their manuscript. Of note, there are no pathognomonic signs for DCM, but rather a constellation of symptoms, physical exam findings, and imaging features that all typically have a slowly progressive course. Imaging findings classically include the absence of a cerebrospinal fluid signal on T2-weighted images, T2 signal hyperintensity, and rarely “snake eyes appearance”, with symmetric circular foci in the gray matter. Additionally, DCM tends to involve progressive neurological deterioration amongst 20% to 62% of patients within 3–6 years. On the other hand, Tu et al. [17] comprehensively discussed the physical exam sensitivity and specificity, commonly used radiographic measures, and T1 vs. T2 MRI findings. Tu et al. also comprehensively summarized the associated genetic polymorphisms, impact of microbiome and molecular features involved in the pathogenesis of disc degeneration, SC dysfunction, axonal injury, and the role and impact of various cell lines on disease course. This study highlighted multiple molecular and micro-structural knowledge gaps, as well as the limited methods to assess degenerative cervical myelopathy appropriately and extensively.

Recognizing the limitations and variability of current outcome measures utilized to study DCM, Soufi et al. [4], assessed the number, quality, and variety of outcome measures currently used in the literature through a systematic review on 148 studies. A total of 39% percent of studies utilized single outcome measures with an average of 2.36 outcome measures used in the studies, with no studies specifically assessing key functions including dorsal column sensory pathway or respiratory, bowel, and sexual function. Objective physical testing of neurological function was rarely utilized, with questionnaires representing 92% (320/349) of all outcome measures utilized, emphasizing the need for a concerted effort in more accurately quantifying neurological dysfunction in DCM, for the purpose of improving diagnosis, measuring severity, and monitoring patients for deterioration. 

It was the intention of this Special Issue to address a wide range of topics regarding DCM and SCI. This project was pursued by the Journal of Clinical Medicine Editorial Board with the hope of contributing new research to help tackle these two prevalent and disabling clinical disorders. We would like to thank the various authors and peer-reviewers for helping to amass this unique body of work (Table 1).

## Figures and Tables

**Table 1:** Summary of published papers in this Special Issue.

Authors	Purpose	Study Design	Main Results	Conclusions
Jentzsch et al. [9]	Investigate whether baseline MRI features predicted the clinical course of the disease utilizing the prospective North American Clinical Trials Network (NACTN) registry	Prospective observational	There were more adverse events in patients with SC signal change (230 (65.0%) vs. 47 (44.8%), *p* < 0.001; odds ratio (OR) = 2.09 (95% confidence interval (CI) 1.31–3.35), *p* = 0.002). The length of stay was longer in patients with SC signal change (13.0 (IQR 17.0) vs. 11.0 (IQR 14.0), *p* = 0.049) and there was no difference between the groups in mortality.	MRI SC signal change may predict adverse events and length of hospital stay.
Oughourlian et al. [5]	Investigate the role of sex differences on the structure of the cerebral cortex in DCM and determine how structural differences may relate to clinical measures of neurological function.	Cross-sectional cohort study	Males demonstrated a significant positive correlation between grey matter volume (GMV) and mJOA score, in which patients with worsening neurological symptoms exhibited decreasing GMV primarily across somatosensory and motor related cortical regions. Females exhibited a similar association, across a broader range of cortical areas including those involved in pain processing. In sensorimotor regions, female patients consistently showed smaller GMV compared with male patients, independent of mJOA score.	Results from the current study suggest strong sex-related differences in cortical volume in patients with DCM, which may reflect hormonal influence or differing compensation mechanisms.
Wolf et al. [6]	Hypothesized that we could reproduce similar patterns of spinal cord motion at the different levels of cervical stenosis among DCM patients presenting with monosegmental stenosis.	Monocentric, prospective, matched-pair-controlled study	Age and severity of stenosis did not relate to spinal cord motion. Spinal cord motion was focally increased at a level of stenosis among patients with stenosis at C4/C5 (*n* = 14), C5/C6 (*n* = 33), and C6/C7 (*n* = 10) (*p* < 0.033). Gender was a significant predictor of higher spinal cord dynamics among men with stenosis at C5/C6 (*p* = 0.048) and C6/C7 (*p* = 0.033).	Gender-related effects lead to dynamic alterations among men with stenosis at C5/C6 and C6/C7. The missing relation of motion to severity of stenosis underlines a possible additive diagnostic value of spinal cord motion analysis in DCM
Sommaruga et al. [11]	Investigate differences in surgical outcomes between SA (stand-alone zero-profile implants) and CP (cervical plating) in ACDF	Retrospective Case series	No significant difference in neurological outcome or rates of dysphagia between SA and CP, and that both lead to overall improvement of symptoms (NDI).	Two approaches have comparable outcomes. Further clinical studies needed to assess.
Wincek et al. [12]	Investigated the long-term effect of the rTMS protocol at frequencies ranging from 20 to 25 Hz and a stimulus strength that was 70–80% of the resting motor threshold in patients with C2–Th12 iSCI	Prospective Cohort Study	The application of rTMS at 20–25 Hz reduced spasticity in the upper extremity muscles, improved the recruitment of motor units in the upper and lower extremity muscles, and slightly improved the transmission of efferent neural impulses within the spinal pathways in patients with C2–Th12 iSCI	Results support the hypothesis about the importance of rTMS therapy and possible involvement of the residual efferent pathways including propriospinal neurons in the recovery of the motor control of iSCI patients
Kadanka et al. [13]	To assess the prevalence and cause of vertigo in patients with degenerative cervical myelopathy (DCM)	Retrospective cross-sectional observational study	Symptoms of vertigo were described by 18 patients (47%) of patients. Causes of vertigo included: orthostatic dizziness in eight (22%), hypertension in five (14%), benign paroxysmal positional vertigo in four (11%) and psychogenic dizziness in one patient (3%).	Despite the high prevalence of vertigo in DCM, the etiology in all cases could be attributed to causes outside cervical spine and related nerve structures.
Kadanka et al. [7]	Assess 10 m walk and run test capability of detecting early gait impairment in a non-myelopathic degenerative cervical cord compression (NMDCC)	Cross-sectional observational cohort study	Walking/running time/velocity, number of steps and cadence of walking/running were recorded; analysis disclosed abnormalities in 66.7% of NMDCC subjects. More significant differences in DCM patients	Standardized 10 m walk/run test has the capacity to disclose locomotion abnormalities in NMDCC subjects.
Ost et al. [10]	To evaluate the current state of a computational models such as Spinal Cord Toolbox (SCT) automated process	Image Analysis Study	Metrics extracted from these automated methods are insufficient to reliably predict disease severity	Although modeling techniques are still in their infancy, future models of DCM severity could greatly improve automated clinical diagnosis and outcomes.
Wilson et al. [8]	Define effects of age and frailty on outcomes following surgical intervention for DCM.	Ambispective	Age and frailty have a significant effect on all outcomes, but the MFI-5 has the largest effect size. Increasing frailty correlated significantly with the risk of perioperative adverse events, longer hospital stay, and risk of a non-home discharge destination.	Measures of frailty have a greater effect size and a higher discriminative value to predict adverse events than age alone.
Gaffari- Rafi et al. [14]	Critically evaluate evidence regarding the role of MRI to influence decision-making and outcomes in acute SCI.	Systemic Review	A total of 32 studies were identified and consistently concluded that MRI was useful prior to surgical treatment (13 studies) and after surgery to assess decompression (two studies), but utility before/after closed reduction of cervical dislocations was unclear (three studies).	MRI is safe and frequently identifies findings alter clinical management in acute SCI, although direct evidence of its impact on outcomes is lacking
Yang et al. [15]	Assess the reporting of study design and characteristics in multi-level degenerative cervical myelopathy (DCM) treated by posterior surgical approaches	Systemic Review	Laminoplasty was described in 56 studies (75%), followed by laminectomy with (36%) and without fusion (16%). Most studies were conducted in Asia (84%), in the period of 2016–2019 (51%), of which laminoplasty was studied predominantly. Twelve (16%) prospective studies and 63 (84%) retrospective studies were identified.	Heterogeneity in the reporting of study and sample characteristics exists, as well as in clinical and radiographic outcomes, with a paucity of studies with a higher level of evidence. Future studies are needed to elucidate the clinical effectiveness of posterior surgical treatments.
Soufi et al. [4]	Assess the neurological, functional, and quality of life (QoL) outcome measures currently in use to quantify impairment in DCM	Systemic Review	The most commonly used instruments were subjective functional scales including the Japanese Orthopedic Association (JOA) (71 studies), modified JOA (mJOA) (66 studies), Neck Disability Index (NDI) (54 studies), and Nurick (39 studies). A total of 92% (320/349) of all outcome measures were questionnaires, whereas objective physical testing of neurological function (strength, gait, balance, dexterity, or sensation) made up 8% (29/349). Studies utilized an average of 2.36 outcomes measures, while 58 studies (39%) utilized only a single outcome measure.	Clinical decision-making and future clinical studies in DCM should employ a combination of subjective and objective assessments to capture the multitude of spinal cord functions to improve clinical management and inform practice guidelines.
Lannon et al. [16]	Summarize current clinical understanding of presentation, pathophysiology, diagnosis, natural history, and surgical management for DCM	Narrative Review	DCM is a common clinical entity with increasing prevalence. Patients with clinically progressive myelopathic symptoms and correlating radiographic evidence of cord compression should be referred for surgical evaluation if it is within the patient’s care goals to prevent further neurologic deterioration	Early diagnosis and surgical management may improve neurologic and overall, outcomes for these patients and, importantly, prevent progressive deterioration
Tu et al. [17]	Discuss epidemiological, diagnostic, pathophysiological, risk factors, molecular features, treatment, and future directions in the management of DCM	Narrative Review	The pathophysiology of the disease is not completely understood, and several mechanisms have been postulated to explain it. The key for successfully treating DCM could be partly J. Clin. Med. 2021, 10, 1214 18 of 25 hidden in the huge array of interactions that take place and have been mentioned in our review.	Given the fact that the aged population in the world is continuously increasing, DCM is posing a formidable challenge that needs urgent attention.
